# Binge drinking leads to an oxidative and metabolic imbalance in skeletal muscle during adolescence in rats: endocrine repercussion

**DOI:** 10.1007/s13105-023-00983-z

**Published:** 2023-09-07

**Authors:** Inés Romero-Herrera, Fátima Nogales, Javier Diaz-Castro, Jorge Moreno-Fernandez, María del Carmen Gallego-Lopez, Julio J. Ochoa, Olimpia Carreras, María Luisa Ojeda

**Affiliations:** 1 Department of Physiology, Faculty of Pharmacy, Seville University, n° 2, 41012 Seville, Spain; 2 Institute of Nutrition and Food Technology “José Mataix Verdú”, University of Granada, Avenida del Conocimiento s/n, 18071 Armilla, Granada Spain; 3 Department of Physiology, University of Granada, Granada, Spain

**Keywords:** Binge drinking, Adolescence, Skeletal muscle, Myokines, Insulin resistance

## Abstract

Binge drinking (BD) is an especially pro-oxidant model of alcohol consumption, mainly used by adolescents. It has recently been related to the hepatic IR-process. Skeletal muscle is known to be involved in insulin action and modulation through myokine secretion. However, there is no information on muscle metabolism and myokine secretion after BD exposure in adolescents. Two experimental groups of adolescent rats have been used: control and BD-exposed one. Oxidative balance, energy status and lipid, and protein metabolism have been analyzed in muscle, together with myokine serum levels (IL-6, myostatin, LIF, IL-5, fractalkine, FGF21, irisin, BDNF, FSTL1, apelin, FABP3, osteocrin, osteonectin (SPARC), and oncostatin). In muscle, BD affects the antioxidant enzyme balance leading to lipid and protein oxidation. Besides, it also increases the activation of AMPK and thus contributes to decrease SREBP1 and pmTOR and to increase FOXO3a expressions, promoting lipid and protein degradation. These alterations deeply affect the myokine secretion pattern. This is the first study to examine a general myokine response after exposure to BD. BD not only caused a detrimental imbalance in myokines related to muscle turnover, decreased those contributing to increase IR-process, decreased FST-1 and apelin and their cardioprotective function but also reduced the neuroprotective BDNF. Consequently, BD leads to an important metabolic and energetic disequilibrium in skeletal muscle, which contributes to exacerbate a general IR-process.

**Supplementary Information:**

The online version contains supplementary material available at 10.1007/s13105-023-00983-z.

## Introduction

Binge drinking (BD) is an acute ethanol (EtOH) consumption model, which brings blood alcohol concentration (BAC) to 0.08 % or higher within 2 h [43]. It has become the most common alcohol consumption pattern used during adolescence [5, 42], a stage especially vulnerable to growth endocrine signals and to the toxic effects of EtOH [2, 41, 45, 47, 48, 64–66] predisposing adolescents to future adult metabolic damage [45, 49]. These detrimental effects are due in part to the fact that BD is a particularly pro-oxidant alcohol consumption pattern. It leads to the production of a wide range of reactive oxygen species (ROS) via the microsomal ethanol oxidizing [1, 36]. It also affects the endogenous antioxidant enzyme system in several tissues [47, 48, 64]. This disruption in oxidative balance results in oxidative stress (OS), which compromises, among others, the mitochondrial function, inflammation, and the apoptosis process [47, 48, 64]. OS has also recently been related to a metabolic deregulation process which affects the cellular energy homeostasis mainly in the liver [29]. Specifically, during adolescence, OS generated by BD consumption alters hepatic energy homeostasis through modulating two important regulators of energy metabolism: the NAD+-dependent sirtuin deacetylase (SIRT1) and AMP-activated protein kinase (AMPK) [45]. Both of them restore cellular energy balance by stimulating diverse catabolic processes [7]; therefore, their decrease in liver predisposes to disorders such as insulin resistance (IR) [17, 45, 54]. Lately, IR process is having higher prevalence in adolescents [20] and highly increases the risk of the development of chronic disease over time [55]. For this reason, it is crucial to know what is happening after BD exposure in other insulin responsive tissues, such as skeletal muscle (SKM), which is a primary site for glucose disposal and storage. However, despite chronic-EtOH consumption clinically affects SKM, leading to alcoholic myopathy [53], which it is characterized by muscle atrophy [52], alcohol effects on IR in SKM still remain controversial [28, 30].

When EtOH is consumed acutely during adulthood, it could lead to a breakdown of SKM fibers [62], deeply affecting SKM protein metabolism. It impairs global SKM protein synthesis by a reduction in the mammalian target of rapamycin complex 1 (mTORC1) pathway [32, 69], but it also increases SKM protein degradation by the ubiquitin-proteasome and by the autophagic-lysosomal systems, being this last pathway more profoundly affected [26]. Since muscle proteins not only have a contractile function but they are also a metabolic reserve of amino acids to support tissues’ energetic needs, this catabolic effect clearly has body energy repercussions [26, 68]. The OS and AMPK phosphorylation processes play important roles in protein turnover [22, 31, 63]. It is described that OS mediates EtOH-induced SKM mitochondrial dysfunction and dysregulates not only the protein synthesis and autophagy [31] but also the catabolic ubiquitin-proteasome pathway [27]; these perturbations are reversed by antioxidant treatments. Additionally, in SKM, it has been reported that AMPK activation is directly dependent on the generation of OS, which increases its catabolic actions [70].

Finally, SKM has been identified as a direct endocrine organ. It releases cytokines and other regulatory peptides which exert paracrine, autocrine, and endocrine effects; they are classified as “myokines” [50]. Recently, novel myokines have been identified, and it has been proved that they may influence metabolism in virtually all organs of the body. Since some of these myokines are induced by SKM contraction and are responsible for mediating energy supply in relation to exercise, they are involved in muscle proliferation. Indeed, they also mediate muscle–organ crosstalk to the brain, adipose tissue, bone, liver, gut, pancreas, vascular bed, and skin in order to balance body energy status [59]. Moreover, their dysregulation is related to metabolic diseases [9, 25] and OS [38]. At the moment, there are just a few studies which analyze several myokines status after chronic alcohol consumption [19], and no data exist after acute one.

The aim of this study is to describe in SKM the effects of BD exposure during adolescence on oxidative and energy balance and their relationship to IR, protein reserves, and myokines secretion.

## Materials and methods

### Animals

In this experimental protocol, twelve adolescent male Wistar rats (Centre of Production and Animal experimentation, Vice-rector’s Office for Scientific Research, University of Seville) were used. Rats were received at 21 days old and housed in groups of two rats per cage for 1 week in order to acclimatize them to the housing conditions and handling. The experimental treatment was conducted over a 3-week period, beginning when the rats reached postnatal day (PND) 28 and ending at 47 days of age. This period corresponds to adolescence in Wistar rats [16]. The animals were kept at an automatically controlled temperature (22–23 °C) and in a 12-hour light-dark cycle (09:00 to 21:00). Animal care procedures and experimental protocols were performed in accordance with EU regulations (Council Directive 86/609/EEC, November 24th, 1986) and approved by the Ethics Committee of the University of Seville (CEEA-US2019-4).

On PND 28, when the adolescent period began, rats were randomly assigned into two groups (*n* = 6/group) according to their treatments: control group (C): rats were given control diet and drinking water ad libitum, and on the corresponding days, an isotonic saline solution (SSF) intraperitoneally (i.p.); binge drinking group (BD): rats were given control diet and drinking water ad libitum, and on the corresponding days, an ethanol solution 20% (v/v) in isotonic saline (3 g/Kg/d) i.p. We have previously proven that with this BD experimental model in adolescent Wistar rats BAC was nearly 125.0 mg/dL [44]. In addition, a standard pellet diet (LASQCdiet® Rod14-R, Märkische, Germani) was available *ad libitum* in all the experimental groups.

### Nutritional control

Body weight and the amount of food consumed by rats were monitored daily until the end of the experimental period. The amount of food ingested every day was calculated by measuring this parameter every morning and the next day; the difference between them was the amount consumed. All measurements were taken at 9:00 a.m. to avoid changes due to circadian rhythms.

### Ethanol treatment

Alcohol BD-exposed group (BD) received a i.p. injection of alcohol (20 % v/v) in SSF (3 g/Kg/d). Alcohol injections were given starting at 7:00 p.m., when the dark cycle began, for 3 consecutive days each week for 3 weeks. No i.p. injections were given during the remaining 4 days of each week (Nogales et al., 2014). Control group (C) received an i.p. injection of an equal volume of SSF at the same time as the alcohol BD-exposed group’s injections.

### Samples

At the end of the experimental period (PND 47), the rats were fasted for 12 h. Then, 24 h after their last EtOH exposure or treatment with saline solution, adolescent rats were anesthetized with an i.p. injection of 28% w/v urethane (0.5 ml/100 g of body weight). The blood was obtained by heart puncture and collected in tubes. The serum was prepared using low-speed centrifugation for 15 min at 1300 × g. Posteriorly, SKM gastrocnemius was removed from the right leg as quickly as possible and immediately frozen in liquid nitrogen and stored at −80 °C. The SKM somatic index (SI) was calculated as SKMSI% = SKM weight/total rat weigh.

### Biochemistry parameter analysis

The serum levels of the creatinine and CPK were measured with an automated analyzer (Technicon RA-1000, Bayer Diagnostics, Leverkusen, Germany).

### Antioxidant enzymes and oxidative stress markers

In order to measure the activity of antioxidant enzymes (SOD, CAT and GPx) as well as the lipid and protein oxidation, SKM tissue samples were homogenized (100 × g for 1min, 1:4 w/v) using a Potter homogenizer (Pobel 245432, Madrid, Spain) in a sucrose buffer (15 mM Tris/HCl, pH 7.4, 250 mM sucrose, 1 mM EDTA, and 1 mM dithiothreitol) in an ice bath. The homogenate was centrifuged at 900 × g for 10 min at 4 °C. The activity of SOD (U/mg protein) was determined by the Fridovich method, which is based on the ability of SOD to inhibit the reduction of cytochrome *c* induced by the xanthine-xanthine oxidase system; it is measured by the absorbance increase at 550 nm for 3 min, due to the reduction of cytochrome *c* by adding xanthine oxidase [15]. Catalase (CAT) activity (U/mg protein) was determined using H_2_O_2_ as substrate by the assay of Beers and Sizer [4], where the disappearance of H_2_O_2_ was followed spectrophotometrically at 240 nm for 3 min. The GPx activity (mU/mg) was determined using the method described by Lawrence and Burk [33]. In this assay, the oxidized glutathione (GSSG) formed by the action of GPx is coupled to the reaction that catalyzes the glutathione reductase (GR) enzyme, measuring the absorbance decrease at 340 nm for 3 min due to the oxidation of NADPH. In order to measure the OS markers in SKM, the amount of lipid peroxidation was evaluated by the quantification of malondialdehyde (MDA) (mol/mg protein), the end-product of oxidative degradation of lipids, using a colorimetric reaction with thiobarbituric acid (TBA) at 535 nm as described by Draper and Hadley [14]. The protein oxidation was measured by the detection of carbonyl groups (CG) (nmol/mg protein) at 366 nm by the method described by Reznick and Packer [56], where the reaction of 2,4-dinitrophenylhydrazine (DNPH) with CG takes place.

### Proteins immunoblotting assays

The expression of the SKM proteins total AMPK (tAMPK), phospho AMPK (pAMPK), SIRT1, IRS-1, mTORC, phospho mTORC (pmTOR), FOXO3a, UKL1, Atrogin-1/MAFBX, and SREBP1 in adolescent rats was conducted using the Laemmli method. The samples utilized contained 60 μg of protein. Proteins were separated on a polyacrylamide gel and were transferred onto a nitrocellulose membrane (BioRad CA, USA) using a blot system (Transblot, BioRad Madrid, Spain). Nonspecific membrane sites were blocked for 1 h with a blocking buffer: TBS (50 mM Tris-HCl1, 150 mM NaCl1, 0.1% (v/v) Tween 20, pH 7.5) and milk powder 3% (BioRad Madrid, Spain), and thereafter, they were probed overnight at 4 °C with the following specific primary antibodies: SIRT1, pAMPKα, and IRS-1 (Cell Signaling Techonology); and tAMPKα, SREBP1, mTOR, pmTOR, FOXO3a, UKL1, and Atrogin-1/MAFBX (Santa Cruz Biotechnology Heidelberg, Germany). Then, membranes were incubated with secondary antibodies (antirabbit or antimouse IgG HRP conjugate, BioRad, Madrid, Spain). Monoclonal mouse anti-GAPDH (Santa Cruz Biotechnology) was used as a loading control, followed by the secondary antibody antimouse IgG Peroxidase conjugate (A9044, Sigma-Aldrich, Spain). The membranes were incubated for 1 min with the commercial developer solution Luminol ECL reagent (GE Healthcare and Lumigen, Buckinghamshire, UK) and analyzed with the Amersham Imager 600 (GE Healthcare, Buckinghamshire, UK). Then, the quantification of the blots was performed by densitometry with the ImageJ program. The results were expressed as percent arbitrary relative units, referring to values in control animals which were defined as 100%.

### Myokines determination

Serum myokine levels, such as IL-6, myostatin, LIF, IL-5, fraktalkine (CX3CL1), FGF21, irisin, BDNF, FSTL1, apelin, FABP3, osteocrin, osteonectin (SPARC), and oncostatin, were measured using MILLIPLEX® Rat Myokine Panel (Millipore Corp., St. Charles, MO, USA), based on immunoassays on the surface of fluorescent-coded beads (microspheres), following the specifications of the manufacturer (50 events per bead, 50 μL sample, gate settings of 8000–15000, time out of 60 s, melatonin bead set of 34). The plate was read on a LABScan 100 analyzer (Luminex Corp., Austin, TX, USA) with xPONENT software for data acquisition. Average values for each set of duplicate samples or standards were within 15% of the mean. Myokines concentrations in plasma samples were determined by comparing the mean of duplicate samples with the standard curve for each assay.

### Statistical analysis

The results are expressed as means ± standard error of the mean (SEM). The data were analyzed using a statistical program Statistica Version 12.0 (StatSoft, Tulsa, OK, USA). To assess difference between C and BD groups, the non-parametric Mann–Whitney–Wilcoxon test was used. The statistical significance was established at *p* < 0.05.

## Results

The results showed that BD exposure during adolescence leads to a lower body weight (*p* < 0.05), without significantly affecting SKM mass (Table 1). Regarding SKM, protein concentration, serum creatinine, and CPK levels remained all of them invariable.

**Table 1:** Morphological characterization of C and BD adolescent rats, SKM profile

	C	BD
Increased body weight (g/d)	5.9 ± 0.1	5.2 ± 0.2*
Food intake (g/d)	15.2 ± 0.4	14.6 ± 0.1
Cranium-caudal length (cm)	16.9 ± 0.3	18 ± 0.7
SKMSI (g wet tissue/g body weight (%))	0.68 ± 0.014	0.67 ± 0.013
Proteins in muscle (mg/ml)	5.02 ± 0.24	4.7 ± 0.18
Serum creatinine (mg/dl)	0.25 ± 0.015	0.26 ± 0.005
Serum CPK (UI/l)	2245 ± 87	2391 ± 96

SKMSI: skeletal muscle somatic index. The results are expressed as mean ± SEM and analyzed by nonparametric Mann Whitney Wilcoxon test. The number of animals in each group is six. Groups: C: control group, BD: binge drinking group. Statistic difference between groups was expressed as *p* value: C vs BD: *p < 0.05

**Fig. 1:**
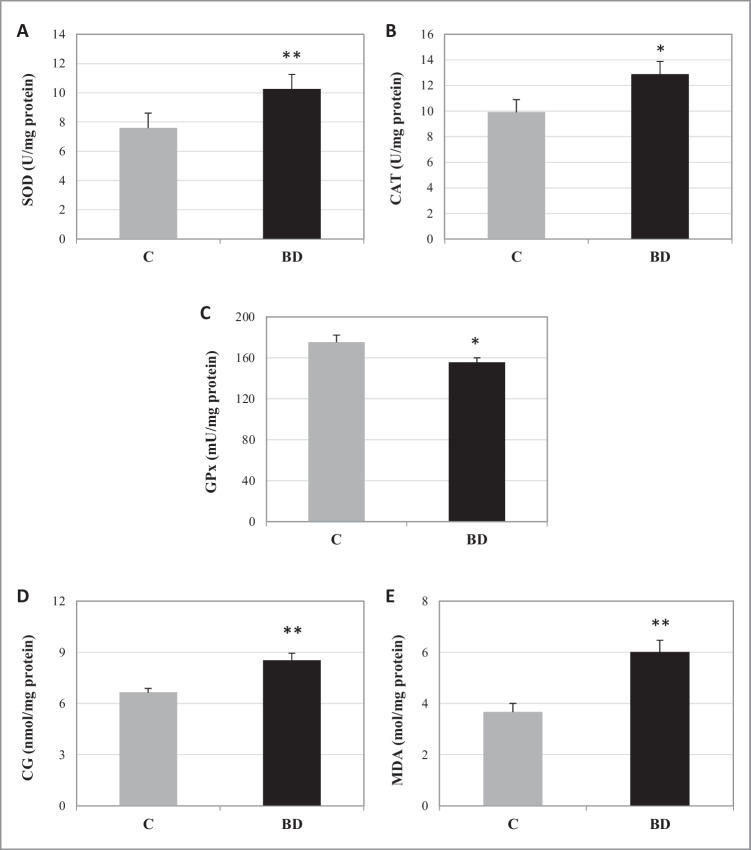
Skeletal muscle oxidative balance after BD exposure. **A** Superoxide dismutase (SOD). **B** Catalase (CAT). **C** Glutathione peroxidase (GPx) enzymes. **D** Oxidation of protein (carbonil group (CG) levels). **E** Lipid peroxidation (malondialdehyde (MDA) levels). The results are expressed as mean ± SEM and analyzed by nonparametric Mann–Whitney–Wilcoxon test. The number of animals in each group is six. Groups: C: control group, BD: binge drinking group. Statistic difference between groups was expressed as *p* value: C vs BD: *p < 0.05, **p < 0.01

Figure 1 shows that adolescent rats exposed to BD present a significant increase in SOD and CAT activities (*p* < 0.01 and *p* < 0.05, respectively) and a decrease in GPx activity (*p* < 0.05), which lead to an oxidative lipid and protein profile (*p* <0.01).

**Fig. 2:**
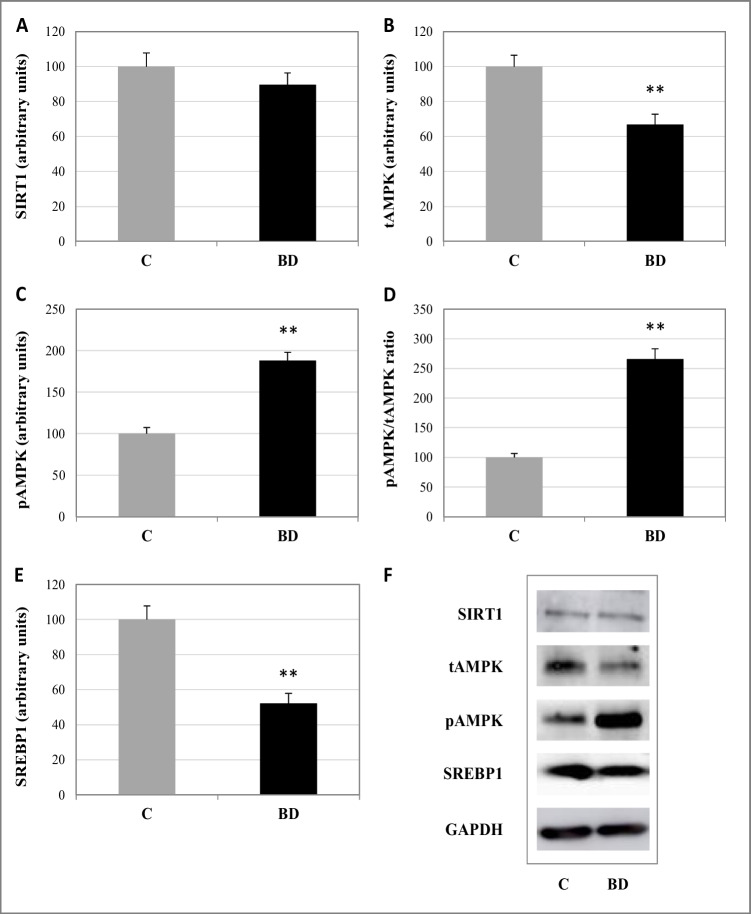
Expression of main energetic regulators in skeletal muscle. **A** SIRT1. **B** tAMPK. **C** pAMPK. **D** AMPK activation ratio. **E** Expression lipogenic transcription factor SREBP. **F** Representative western blots of proteins (normalized to GAPDH). The results are expressed as mean ± SEM and analyzed nonparametric Mann–Whitney–Wilcoxon test. The number of animals in each group is six. Groups: C: control group, BD: binge drinking group. Statistic difference between groups was expressed as *p* value: **p < 0.01

**Fig. 3:**
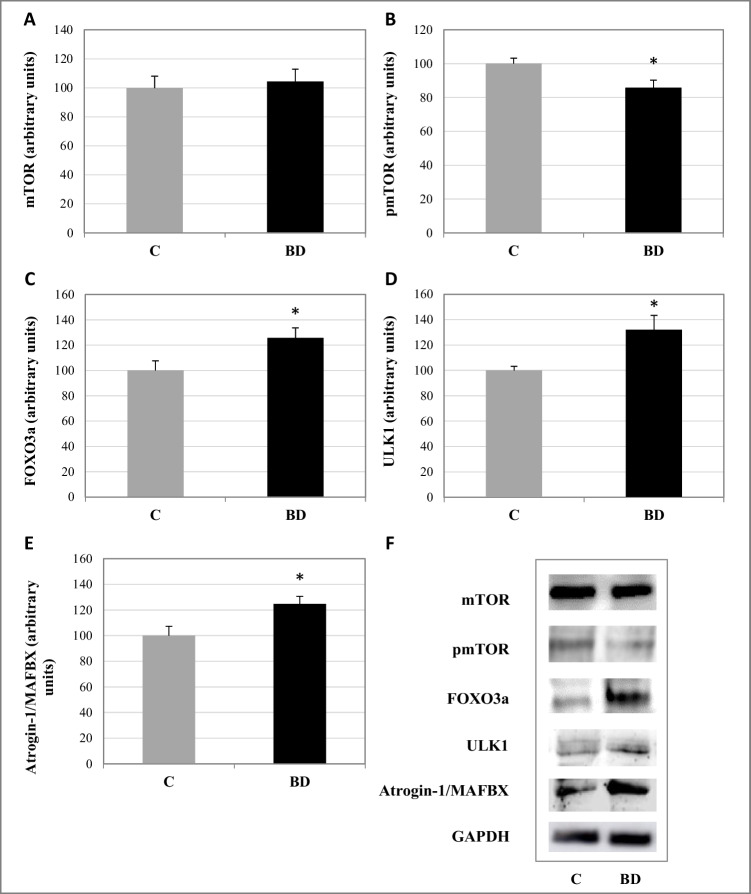
Expression of main transcription factors implicated in protein metabolism of skeletal muscle: **A** mTOR. **B** pmTOR. **C** FOXO3a. **D** ULK1. **E** Atrogin-1/MAFBX. **F** Representative western blots of proteins (normalized to GAPDH). The results are expressed as mean ± SEM and analyzed by nonparametric Mann–Whitney–Wilcoxon test. The number of animals in each group is six. Groups: C: control group, BD: binge drinking group. Statistic difference between groups was expressed as *p* value: *p < 0.05

Respect to energy marker proteins (Fig. 2), BD exposure does not affect SIRT1 expression and decreases tAMPKα expression (*p* < 0.01). However, alcohol consumption activates AMPK function since it increases pAMPKα expression and thus pAMPK/tAMPK ratio (*p* < 0.01). This increase is in concordance with the lower SREBP1 expression values found after BD exposure (*p* < 0.01). Furthermore, protein turnover is significantly affected by BD (*p* < 0.05), since pmTOR expression decreased whereas FOXO3a expression increased (*p* < 0.05) (Fig. 3). In this context, Atrogin-1/MAFBX and ULK1, both involved in protein degradation, were increased (*p* < 0.05).

**Fig. 4:**
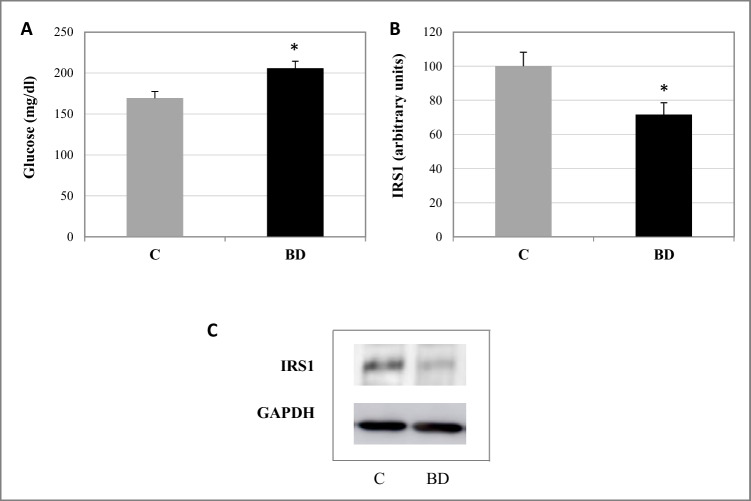
Serum glucose concentration (**A**) and expression of IRS1 in skeletal muscle (**B**). **C** Representative western blots of IRS1 (normalized to GAPDH). The results are expressed as mean ± SEM and analyzed by nonparametric Mann–Whitney–Wilcoxon test. The number of animals in each group is six. Groups: C: control group, BD: binge drinking group. Statistic difference between groups was expressed as *p* value: *p < 0.05

In adolescent rats, glucose serum levels were significantly increased after BD exposure (*p* < 0.05) (Fig. 4); however, IRS-1 expression in SKM decreased (*p* < 0.05). To conclude, BD exposure during adolescence significantly alters myokines serum values (Table 2). BD not only increases IL-6 and myostatin (p < 0.05) serum levels but it also decreases LIF (*p* < 0.05), CX3CL1 (*p* < 0.01), FGF21 (*p* < 0.01), irisin (*p* < 0.01), BDNF (*p* < 0.01), FSTL-1 (*p* < 0.01), apelin (*p* < 0.01), and SPARC (*p* < 0.05) values.

**Table 2:** Effects of BD exposure on 14 serum myokines levels and their main functions

Myokine	C	BD	Main function
IL-6	0.31 ± 0.006	0.41 ± 0.02*	↑Muscle atrophy and fatty acid oxidation ↑Lipolysis and adipose browning ↑Insulin secretion, glucose liberation by liver, and glucose uptake by muscle ↓apetite
Myostatin	393.2 ± 2.9	414 ± 6.7*	↑Muscle atrophy, ↓muscle mass, ↓bone formation
LIF	7.2 ± 0.3	5.3 ± 0.2*	↑Proliferation of myoblast
IL-15	1.09 ± 0.04	1.14 ± 0.04	↑Myoblast differentiation and fat metabolism Antiviral
Fractalkine (CX3CL1)	39.7 ± 0.4	28.8 ± 0.9**	Protects muscle from IR: ↑glucose uptake Protects beta-cell from inflammation
FGF21*	3.5 ± 0.15	2.1 ± 0.12**	↑Muscle mass and mitochondria biogenesis ↑Glucose uptake and adipose browning
Irisin	205 ± 5	146 ± 14**	↑Muscle mass, hypertrophy and fatty acid oxidation ↑Glucose uptake and adipose browning
BDNF	3527 ± 135	2662 ± 181**	↑Muscle regeneration and fatty acid oxidation ↑Hippocampal neurogenesis (memory, learning)
FSTL-1*	2571 ± 71	1949 ± 30**	↑Endothelial function and revascularization ↑Cardioprotection
Apelin	106.9 ± 1.6	89.2 ± 1.6**	Improves cardiovascular function, blood pressure, angiogenesis, and drinking behavior
FABP3	9022 ± 340	10029 ± 427	Fatty acid carrier, supply energy to heart
Osteocrin	38.8 ± 0.5	38.2 ± 0.8	Regulate bone growth and neuronal function prevents diabetic cardiomyopaty
Osteonectin (SPARC)	4.4 ± 0.2	3.6 ± 0.3*	↑Muscle repair and bone mineralization
Oncostatin (OSM)	0.21 ± 0.02	0.24 ± 0.01	Bone formation and destruction Important in liver development, haematopoeisis, and inflammation

The results are expressed as mean ± SEM and analyzed by nonparametric Mann Whitney Wilcoxon test. The number of animals in each group is six. Groups: C: control group, BD: binge drinking group. Statistic difference between groups was expressed as p value: *p < 0.05; **p < 0.01. The symbols “↑” indicate increase and “↓” indicate decrease. Main functions were extracted from the following revisions: Lee and Jun (2019); Mancinelli et al. (2021); Severinsen et al. (2020), Severinsen and Pedersen (2020); Oliveira dos Santos. (2021); Chen et al. (2020)

## Discussion

### BD exposure does not impact the overall morphological characteristics of skeletal muscle

Despite the fact that EtOH consumption does not change the amount of food intake, BD rats presented a lower increase in body weight, without affecting their length, probably related to a lower adipose mass. However, general parameters relative to SKM anabolism and catabolism processes such as SKMSI, protein muscle content, serum creatinine, and CPK values remained significantly unaffected. Since proteins are the best conserved macromolecules in the body, these effects could be postponed in time. Nevertheless, a tendency to a decrease in SKM protein content seems to be occurring.

### BD exposure leads to oxidative damage in skeletal muscle and to an increase in AMPKα activation, avoiding lipogenesis

**Fig. 5:**
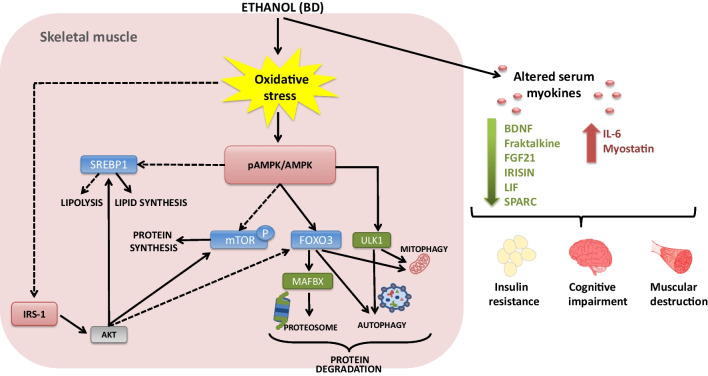
Effect of BD exposure during adolescence on skeletal muscle metabolism and myokine secretion. When EtOH is consumed in large amounts, as in BD exposure, it arrives at other tissues like SKM. This EtOH generates a great amount of reactive oxygen species, which in the end lead to the appearance of oxidative stress. Consequently, the expression ratio of pAMPK/tAMPK increases, resulting in a stimulated AMPK function. The active AMPK, as an important energy status sensor, regulates several other proteins. On the one hand, it inhibits the expression of SREBP1 and mTOR, which, respectively, will decrease the lipid and protein synthesis; on the other hand, AMPK stimulates FOXO3a and ULK1 expressions. FOXO3a will result in the stimulation of the protein breakdown pathways: it promotes the initiation of autophagy; and increasing the Atrogin-1/MAFBX expression, it also activates the proteosome system. Besides, ULK1, together with FOXO3a, will stimulate the initiation of autophagy and the mitochondrial autophagy process (mitophagy). Additionally, the oxidative stress diminishes the expression of IRS-1, which will not stimulate Akt. This protein, when active, stimulates mTOR and inhibits FOXO3a; in this case, being inactive, it will produce the opposite effects, promoting a general IR process. Furthermore, EtOH exposition seriously affects myokine secretion to serum, increasing IL-6 and myostatin levels and decreasing BDNF, fractalkine, FGF21, irisin, LIF, and SPARC values. This altered pattern of secretion contributes to the muscular destruction, the insulin resistance, and to the development of a cognitive impairment. In summary, BD exposition promoted the lipolysis and protein degradation in SKM and inhibited protein synthesis and lipogenesis, also altering the insulin signaling pathway, contributing to a general IR process. Solid lines and hatched lines indicate stimulatory and inhibitory actions, respectively

BD exposure during adolescence changes the antioxidant balance activity in the gastrocnemius muscle (Fig. 5). It leads to an increased activity of SOD and CAT; however, these higher activities generated due to EtOH exposure to combat ROS were not efficient, since lipid and protein oxidation appeared in any case. Therefore, like in serum, liver, kidney, and heart [46–49, 64], BD exposure during adolescence is also a potent pro-oxidant in the gastrocnemius muscle.

OS is deeply related to the energetic cellular balance. BD-OS generated in liver contributes to a decrease in SIRT1 and AMPK activation [45]. However, in SKM, despite the fact that OS appears, SIRT1 expression remains unaffected, and AMPKα activation is enhanced. Previous studies describe that in SKM, AMPK is clearly activated by OS, independently of changes in the AMP/ATP ratio [70].

Moreover, it has been determined in myotubes that an increase in the myokine IL-6 activates AMPKα subunit [8]. In this study, IL-6 serum levels are markedly increased after BD exposure and could be contributing to the AMPK activation. Indeed, several authors have described the effects of IL-6 in SKM fatty acid metabolism in human and in vitro, confirming that it activates lipolysis independently of hormonal signals, increasing rates of fatty acid oxidation and pointing to AMPKα as a possible master key [6].

SREBP1 is a key lipogenic transcription factor which is up-regulated by glucose and insulin and down-regulated by AMPK in SKM, pointing to AMPKα as an effective approach to avoid tissue lipogenesis [23]. In the present study, the higher pAMPK expression found in BD rats is in concordance with the lower SREBP1 values found, indicating that lipolysis is taking place in SKM. This fact coupled with lipid oxidation indicates that lipid homeostasis is definitely damaged in this tissue.

### BD exposure affects protein turnover homeostasis by enhancing oxidative stress and the AMPKα-FOXO3a axis

There are different signaling pathways which connect OS to decreased protein synthesis and increased proteolysis patterns. OS contributes to SKM protein catabolism by increasing the expression of proteins involved in autophagy and the proteasome system of proteolysis; it also activates muscle proteases such as caspases and calpains; and it affects the structure of myofibrillar proteins favoring proteolytic processes. Moreover, it also contributes to depress global SKM protein synthesis by affecting mTOR signaling; however, most of these studies are still in vitro [18, 51]. Finally, OS in SKM leads to AMPKα activation [70].

AMPKα activity in SKM is known to govern protein turnover mainly towards a catabolic state by directly increasing the mammalian autophagy-initiating kinase ULK1 and the transcription factor FOXO3a activities, both related to protein breakdown; but it also acts by decreasing mTOR activity, which is related to protein synthesis [58]. AMPKα also promotes mitochondrial biogenesis to produce more ATP. The AMPKα-ULK1 axis is deeply related not only to autophagy initiation but also to the mitochondrial autophagy (mitophagy) process [21]. The AMPK-FOXO3a axis is a potent myofibrillar proteolytic system that activates the ubiquitin-proteosome system by increasing a muscle specific F-box protein, Atrogin-1, also known as MAFBX, which is a principal component of the SCF ubiquitin ligase complex that ubiquitinates and targets calcineurin for degradation. FOXO3a also activates the autophagosome-lysosome system and the mitophagy process [57]. As a consequence, the higher OS and the pAMPKα levels found in BD rats result in higher FOXO3a, Atrogin-1, and ULK1 levels, and lower pmTORC1 expressions [24]. These results clearly imply that lower protein synthesis and higher protein destruction have taken place after BD exposure. Together with the high protein oxidation found, it is clear that BD exposure during adolescence affects protein metabolism and function in the SKM.

### BD exposure seems to promote insulin resistance in SKM

Therefore, SKM dysfunction during BD exposure contributes to a disruption in lipid and protein homeostasis; moreover, it seems to promote the instauration of the insulin resistance process, since in SKM IRS-1 expression is decreased and hyperglycemia is appearing [16]. It is described that OS in mammalian SKM in vitro cells leads to a loss of IRS-1 protein, in part due to a p38 MAPK-dependent mechanism [3]. Therefore, the OS detected in this study could be contributing to induce IR. The anabolic insulin signaling process activates the phosphoinositide 3-kinase (PI3K)/Akt pathway. Akt not only phosphorylates many proteins such as mTOR and SREBP1, resulting in augmented protein and lipid synthesis [12], but it also has a downstream effect on FOXO3a, avoiding protein degradation [37]. According to that, after BD exposure, pmTORC1 and SREBP1 expressions are decreased, and FOXO3a expression is enhanced, indicating that the insulin-Akt axis is not effectively acting in SKM, which is consistent to an IR instauration.

### BD exposure clearly modulates the myokine secretion pattern associated with insulin resistance

The SKM not only excretes myokines in order to maintain its own homeostasis but it also has a direct influence on the general energy status. Like in hepatic steatosis, where there is an imbalance in myokine, hepatokine, and adipokine secretions, which are implicated in negative metabolic processes [13], in BD-exposed adolescent rats, which present hepatic alcoholic damage [45], it may also happen. Consequently, it is important to know the myokines balance after BD exposure. In this study, for the first time, it is demonstrated that EtOH exposure seriously affects myokine secretion, having important metabolic and energetic repercussions even during adolescence.

In this context, BD exposure during adolescence increased IL-6 and myostatin serum levels, two myokines that increase muscle atrophy, decrease muscle mass, and lead to fatty acid oxidation in muscle by different mechanisms [35, 38]. They are presumably contributing to the occurrence of biomolecular changes in the SKM protein synthesis route described above after BD exposure, promoting protein catabolism. Nevertheless, in order to have macroscopic repercussions, a longer EtOH exposure would probably be needed. Besides, it is also described that myostatin increases ROS generation in SKM through the NF-κB pathway, which is also related to inflammation [67]. Therefore, myostatin could be another pro-oxidant factor in the OS generated in SKM after BD exposure. IL-6 signaling has also been associated with stimulation of lipolysis and adipose browning by the activation of AMPK [71]. In addition, IL-6 mediates a cross-talk between insulin-sensitive tissues, among others by increasing glucose release by liver [13, 59]. The elevated IL-6 values found after BD confirm the hyperglycemia and the lower IRS-1 expression detected, being this myokine contributing to the general BD-IR process.

In contrast, BD exposure decreased SPARC, LIF, CX3CL1, FGF21, irisin, and BDNF serum levels. All of them have anabolic effects on SKM such as the stimulation of the proliferation of myoblast, an increased muscle mass and muscle regeneration, or an increased mitochondria biogenesis, to name but a few [35]. These proteins’ decreases contribute to the SKM catabolic state generated by BD exposure, and difficult SKM remodeling. Moreover, CX3CL1, FGF21, and irisin promote glucose uptake, protect beta-cell from inflammation, increase insulin sensitivity in muscle, and lead to adipose browning; all these effects decrease the IR process [13, 59, 60]. Since they are decreased, IR process is probably taking place, at least in part by the damage generated by BD to SKM. Furthermore, BDNF is involved in neurogenesis and in the protection against oxidative damage and neuronal apoptosis. Its increase promotes synaptic plasticity in hippocampal neurons, neurogenesis, and memory and learning processes [39]. Therefore, the reduced BDNF found in these BD rats may also be associated with the well-known neurotoxic effects of EtOH consumption during adolescence, being many of these effects specific to adolescents and not found in parallel adult studies [10].

Additionally, BD exposure also decreases serum levels of the myokines FST-1 and apelin; both of them are related to the endothelial function, revascularization, cardioprotection, and blood pressure regulation [9, 13, 59, 60]. These results are in agreement with those obtained in a similar BD experimental model by Ojeda et al. [48], where BD exposure led to OS, inflammation, and apoptosis in cardiac myocytes and to an increase in the endothelial markers CTGF, VEGF, tPAI-1, and cav-1, joint to a higher blood pressure and heart rate. These results point to SKM as a master key not only in metabolic and energy balance but also in cardiovascular modulation after BD exposure.

FGF21 and FSTL-1 serum levels are significantly the most affected myokines by BD exposure. Both of them have the liver as a cross-talk organ [9] and are also secreted by liver [13], being considered hepatokines. They have beneficial actions on the hepatic metabolism. Among other functions, FGF21 increases the oxidation of fatty acids in the liver, reverses hepatic steatosis, and increases energy expenditure [13, 34, 40]; in BD-exposed adolescent rats, its reduction is in concordance with the liver steatosis found. FSTL-1 plays a crucial role in liver fibrosis [61], being its decrease related to the liver damage caused by the acute EtOH ingestion [11, 45, 47].

Finally, according to the rats’ final body length, BD exposure has no repercussion on those myokines related to bone remodeling and function, such as osteocrin and oncostatin. As for IL-15 and FABP3 serum levels, they were also unaffected by BD exposure.

Consequently, it has been demonstrated that BD exposure during adolescence clearly disrupts the SKM through the OS generated, affecting the metabolic and energy balance, and the SKM structure and function. These imbalances have deep important endocrine repercussions. In summary, for the first time, it has been demonstrated that BD-SKM damage has general endocrine repercussions via myokines, especially by those related to muscle protein loss, the IR process and glucose uptake, lipolysis, and the adipose browning, affecting the metabolic and energetic body balance and the hepatic health. Furthermore, since BDNF serum levels are reduced, it could be promoting EtOH neurotoxic effects. All in all, these findings support the hypothesis that adolescent BD promotes SKM atrophy, which could lead to long-lasting changes in the adult metabolism, increasing risks for future cardiopathologies.

### Supplementary Information

ESM 1 (PDF 585 kb)
