# Supplementary material for: Binge drinking leads to an oxidative and metabolic imbalance in skeletal muscle during adolescence in rats: endocrine repercussion
Source: J Physiol Biochem. 2023 Sep 7;79(4):799–810. doi: 10.1007/s13105-023-00983-z (PMC10635949; doi:10.1007/s13105-023-00983-z)

COMPLETE WESTERN BLOT MEMBRANE IMAGES

***The samples marked in red are the images shown in the publication graphics. Other samples not named shown in the images belong to other experiments not relative to the publication.**

SIRT1 (100 kDa)

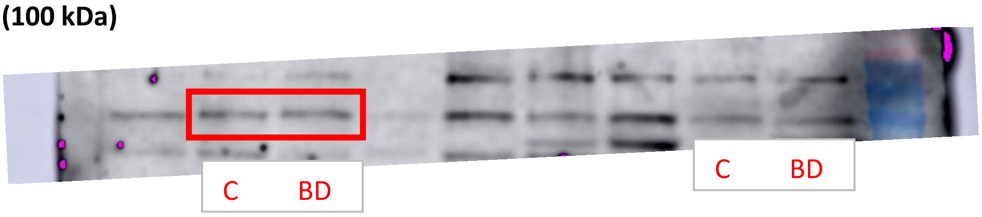

tAMPK (60 kDa)

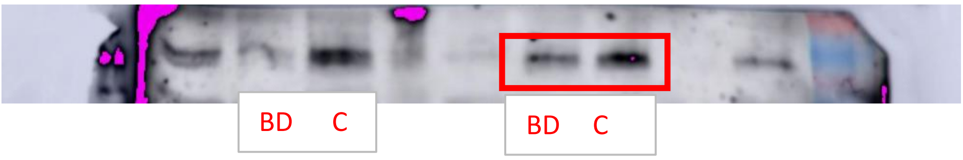

pAMPK (60 kDa)

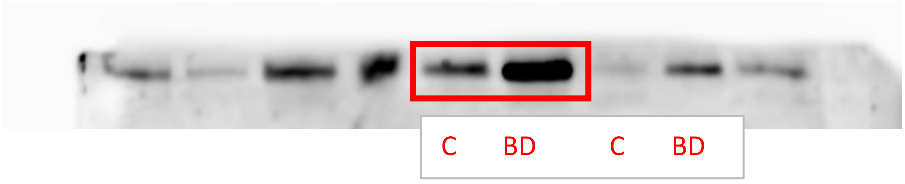

SREBP1 (50 kDa)

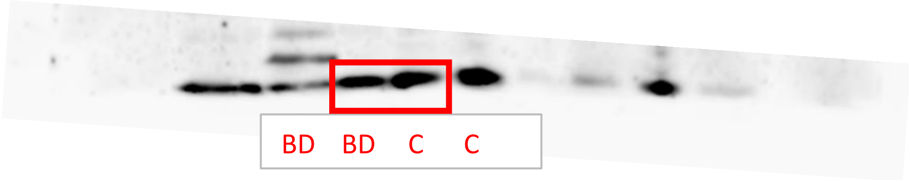

mTOR (245 kDa)

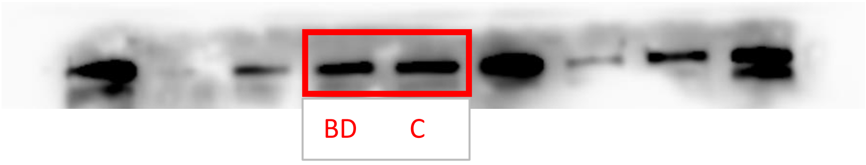

pmTOR (100 kDa)

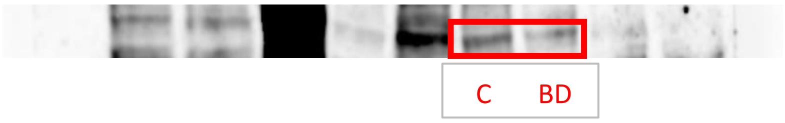

FOXO3A (70 kDa)

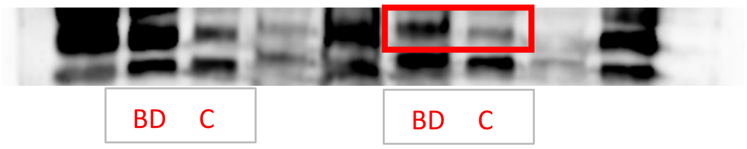

ULK1 (90 kDa)

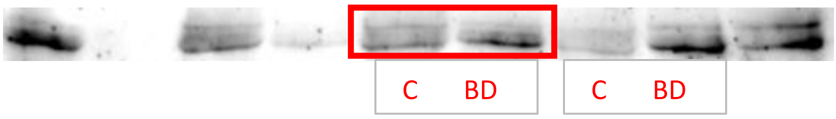

Atrogin-1/MAFBX (35 kDa)

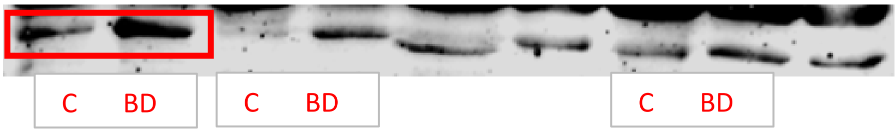

IRS1 (150 kDa)

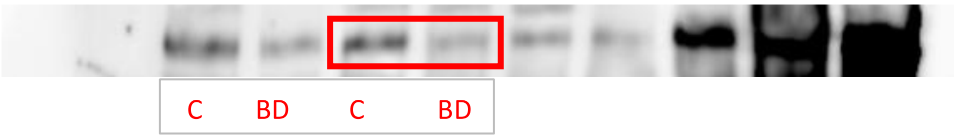

Supplement: Supplementary file 1 — (PDF 585 kb) [file 13105_2023_983_MOESM1_ESM.pdf]
